# *Babesia* Species of Domestic Cats: Molecular Characterization Has Opened Pandora's Box

**DOI:** 10.3389/fvets.2020.00134

**Published:** 2020-03-27

**Authors:** Barend L. Penzhorn, Marinda C. Oosthuizen

**Affiliations:** ^1^Vectors and Vector-borne Diseases Programme, Department of Veterinary Tropical Diseases, Faculty of Veterinary Science, University of Pretoria, Onderstepoort, South Africa; ^2^Research Associate, National Zoological Garden, South African National Biodiversity Institute, Pretoria, South Africa; ^3^Research and Postgraduate Studies, Faculty of Veterinary Science, University of Pretoria, Onderstepoort, South Africa

**Keywords:** *Babesia felis*, *Babesia leo*, *Babesia lengau*, *Babesia canis presentii*, *Babesia hongkongensis*, *Babesia* species cat Western Cape, *Babesia vogeli*, feline babesiosis

## Abstract

This is the first comprehensive review of the literature pertaining to *Babesia* species reported from domestic cats. Description of the four species (*Babesia felis, Babesia cati, Babesia herpailuri*, and *Babesia pantherae*) named based on morphology and/or host specificity is documented. Feline babesiosis is of major veterinary concern only in South Africa. Reports of the rare occurrence of feline babesiosis cases in Europe (France, Germany, Poland, and Spain) and Asia (Israel, India, and Pakistan) are documented. Molecular characterization has revealed that cats can harbor a variety of *Babesia* species. The previous practice of referring to all piroplasms, especially small ones, seen on feline blood smears as *B. felis* is therefore no longer tenable. The near-full-length 18S rRNA gene sequences entered into GenBank in 2001 (accession no. AF244912) are designated as definitive for *B. felis* sensu stricto. All published literature relating to molecular characterization of feline *Babesia* species that could be traced was critically assessed. Four *Babesia* species are now known to be involved in causing feline babesiosis in South Africa: the closely related *B. felis* s.s. and *Babesia leo* (clade I), *Babesia lengau* (clade II), and *Babesia* species cat Western Cape (clade VI, *Babesia* s.s.). Clade VI also includes *Babesia canis presentii* and *Babesia hongkongensis* reported from cats in Asia. Six other *Babesia* species have been reported from domestic cats: the dog-associated *B. canis* s.s., *Babesia gibsoni*, and *B. vogeli*, as well as *Babesia lohae, Babesia microti*, and *Babesia vulpes*. Phylogenetic relationships of all named species were assessed and are presented as trees. The relatively high prevalence of *B. vogeli* in clinically healthy cats (16% in Brazil, 13% on St Kitts, and 8.1% in Portugal) suggests that immunocompetent cats can harbor the infection with no discernible untoward effects. Reports of occurrence of *B. felis* and other *Babesia* species in domestic cats should be accepted only if they are supported by credible molecular provenance.

## Introduction

In the first reference to feline babesiosis, published in 1904, it was claimed that “spontaneous pyroplasmosis” occurred in tame and wild cats in India (1), but no supporting information was provided. This record is dubious, especially since the authors also include humans and chickens in their list of affected hosts. Published literature on feline babesiosis is rather meager, compared to the wealth of papers on babesiosis (or piroplasmosis) in other domestic animals. This is not surprising because babesiosis of domestic cats seems to be of major veterinary concern only in a restricted area of South Africa (2). Sporadic cases diagnosed elsewhere are deemed of sufficient interest to warrant documentation as case reports. All cases documented to date occurred in the Old World.

Before the advent of molecular characterization of species, one approach to identifying *Babesia* species was based on descriptions, detailed or otherwise, of morphology (including dimensions) of the organism. This led to the notion that *Babesia* species can be classified either as “large” or “small.” This approach had its pitfalls because piroplasms undergo marked morphological changes during their development in erythrocytes. In addition, there can be substantial overlap between measurements of “large” and “small” babesias. A second approach was to assume that *Babesia* species are more or less host specific. The logical outcome of such reasoning was that any *Babesia* seen on a blood smear of a wild felid should, by definition, be the same species as that found in domestic cats. Neither approach was satisfactory, and caution should be exercised when quoting names of *Babesia* species used in such publications.

The advent of molecular characterization has led to the description of various new *Babesia* species from domestic cats. A similar situation exists in African great ape populations, for example, where molecular characterization is revealing an unexpected diversity of *Plasmodium* species that had not been detected previously (3, 4).

This is the first comprehensive review of the literature pertaining to *Babesia* species reported from domestic cats, either from clinical cases or as incidental findings. Articles on piroplasms of wild felids are included only if there is a direct connection with domestic cats. In the first section, feline *Babesia* species named based on morphology and/or host-specificity are documented, in chronological order. In the second section reports of clinical feline babesiosis from Southern Africa, Europe and Asia are documented. In the third section, *Babesia* species reported from domestic cats based on molecular characterization are critically evaluated. In this evaluation, the near-full-length 18S rRNA gene sequences, entered into GenBank in 2001 (accession no. AF244912), were regarded as definitive for *Babesia felis* sensu stricto (5). In addition to *B. felis* s.s., four *Babesia* species and one subspecies have been described from domestic cats based on gene characterization. Various other *Babesia* species have also been reported from domestic cats. Data on all of these *Babesia* species were assembled and phylogenetic trees prepared.

## Feline *Babesia* Species Named Based on Morphology and/or Host-Specificity (1929–1972)

The four *Babesia* species involved are discussed in chronological order, based on the date of their description. In 1929, Davis (6) described and named *B. felis* from a wild cat (*Felis ocreata*, presumably *Felis sylvestris*) in Sudan. Trophozoites varied in diameter from less than 1 μm to 2.25 μm, the majority being about 1.25 μm; the four pear-shaped merozoites were arranged in a cruciform manner (6). In the course of a thorough investigation, 22 domestic cats were artificially infected with a few drops of infected blood in 2% sodium citrate solution. Irrespective of route of infection (intravenous, intraperitoneal, or subcutaneous), all cats became parasitemic, but none developed overt clinical signs, even after undergoing splenectomy. Infection with the piroplasm did not precipitate overt clinical signs even after serial passage through five cats.

In February 1933, two cougars (*Puma concolor*) shipped from San Francisco arrived at the Cairo Zoological Gardens, Egypt, where they were housed in close proximity to other large felids (7). Within 2 to 3 weeks, both animals developed clinical signs of babesiosis and succumbed to the disease. Trophozoites resembled those of the canid-associated *Babesia gibsoni* (8), with an obtuse angle between pear-shaped merozoites joined at their tips. These piroplasms were named *Babesiella felis* by Carpano (7), who also argued that the piroplasm described by Davis (6) should be called *Nuttallia felis*. In this, Carpano (7) followed the classification proposed by du Toit (9). Although this case is not specifically linked to domestic cats, it is included because the name chosen for the piroplasm by Carpano (7) could lead to confusion.

A few years later, in 1937, Mangrulkar (10) reported finding small intraerythrocytic piroplasms on blood smears made during an autopsy on a somewhat anemic cat in India. Because merozoite tetrads (Maltese crosses) were not observed, Mangrulkar (10) concluded that the piroplasms concerned were different from *B. felis* and *Babesiella felis*. (*Babesiella felis* is not referred to further in this review; *B. felis* henceforth implies *Babesia felis*.) Since Mangrulkar (10) lacked suitable material for further study, he refrained from naming a new species. In 1950, a similar small piroplasm (0.5–2.5 μm), seen on blood smears made from an apparently healthy Indian wild cat, was named *Babesia cati* (11). Attempts at transmitting this piroplasm to domestic cats were unsuccessful.

In 1967, a “large” *Babesia* from a neotropical jaguarundi (*Puma yagouaroundi*, previously assigned to the genus *Herpailurus*) was established artificially in spleen-intact domestic cats, which did not develop overt clinical signs (12). In 1969, this species was described and named *Babesia herpailuri* (13). In 1972, *Babesia pantherae* from leopards (*Panthera pardus*), a piroplasm smaller than *B. herpailuri*, was established in splenectomized domestic cats (14, 15). A small piroplasm matching the morphological description of *B. felis* was also transmitted artificially from leopards to domestic cats (14).

## Occurrence of Clinical Cases

Clinical cases of feline babesiosis were first reported from the vicinity of Cape Town, South Africa, in 1937. In 1972, Dennig and Brocklesby (14) confirmed that clinical cases had not been reported outside of South Africa.

### Southern Africa

The first two reports of babesiosis in domestic cats appeared in 1937. In the first paper, a case report, the cat was pyrexic and anemic and recovered after administration of quinuronium sulfate (16). Piroplasms had an average diameter of approximately 1.5 μm, but rare pyriforms measured up to 4 μm long. Noting that cats artificially infected with *B. felis* by Davis (6) had not developed overt clinical signs and following the classification of du Toit (9), Jackson and Dunning (16) named the organism incriminated in this case *N. felis* var. *domestica*. The second article was a general report on babesiosis occurring in free-ranging cats (17). Clinical signs included pyrexia and anemia; treatment with quinuronium sulfate or trypan blue affected clinical cure (17). Since the disease only occurred in cats that had access to undisturbed natural vegetation, McNeil (17) speculated that the wild cat (*Felis caffra*; syn. *F. sylvestris*) may be a reservoir of infection. The disease was subsequently reported from Knysna, a coastal town approximately 430 km east of Cape Town (18). Here the cats also tended to be pyrexic.

The first systematic investigation into the disease included 70 cats presented at veterinary clinics and 20 artificially infected cats (19). The causative organism was called *B. felis*. Blood from a sick cat whose owners lived in what is now Table Mountain National Park was used for the 20 artificial infections. Clinical signs in reacting cats included lethargy, anorexia, and anemia, but pyrexia was not a feature of the disease (19). The absence of pyrexia may indicate that the pathogen involved here was not the same as that reported in the earlier clinical cases.

In contrast to earlier reports, trypan blue and quinuronium sulfate were not effective in treating cats artificially infected with *B. felis* sensu lato (19). This is a further indication that more than one piroplasm species may have been involved in earlier reports.

Babesiosis was subsequently shown to occur fairly commonly in cats along the eastern and southern coast of South Africa and adjacent inland areas, primarily in summer (2). It also occurs along the eastern escarpment in Mpumalanga and Limpopo Provinces (20).

A large piroplasm was observed on blood smears of a sick cat from the suburbs of Harare, Zimbabwe (21). The cat had a temperature of 40°C. Treatment with diminazene affected clinical cure. In line with dogma acceptable at the time and based on morphology and measurements, the organism was tentatively identified as *B. herpailuri*.

### Europe

Feline babesiosis is rare in Europe. The first clinical case, reported in 1992, was a somewhat anemic 8-year-old cat in France (22). A low parasitemia of small piroplasms (1.5–2 μm) was found. The report mentioned large piroplasms (3.5–5.5 μm in diameter) on a blood smear of a cat from Paris; no further detail was given. Bourdeau (23) hypothesized that the small organisms were *Babesia divergens* or *Babesia microti*, whereas the large one was *B. canis* or *Babesia vogeli*. A subsequent case from France was attributed to *Babesia vulpes* (referred to as *Babesia annae*) (24).

The only clinical case in Germany, reported in 1997 (25), was a 10-month-old cat imported from northern Sweden. Clinical signs included pyrexia (39.6°C) and anemia. The cat responded well to treatment with imidocarb. Blood smears indicated a large *Babesia*, with mean dimensions 3.6 × 2.3 μm. In Poland, a 10-year-old cat showing weakness, anemia, fever, and hematuria recovered fully after administration of imidocarb (26). Piroplasms on blood smear resembled *B. canis*, but polymerase chain reaction (PCR) sequencing of a 559-bp fragment of the 18S rRNA gene showed only 95% homology with *B. canis* s.s.

On the Iberian Peninsula, *B. canis* s.s. was incriminated in one feline babesiosis case in Spain (27). Subsequently, *B. canis* s.s. was reported from 1.3% and *B. vogeli* from 8.1% of 320 cats presented at 30 veterinary medical centers in northern and central Portugal (28).

### Asia

As mentioned previously, an autopsy on a somewhat anemic cat in India indicated the presence of small piroplasms (10). Also in India, small amoeboid, oval, or rounded parasites of varying sizes resembling *B. felis* were seen in blood smears from a pyrexic, lethargic, anorexic kitten that made an uneventful recovery after administration of primaquine phosphate (29). A large babesia, incriminated in causing disease in a cat in India, was successfully treated with diminazene (30).

In Lahore, Pakistan, babesiosis was diagnosed in 163 (3.1%) of cats presented at a university clinic (31). Blood smears from 50 cats in Mosul, Iraq, were examined for presence of hemoparasites (32). It is not clear whether the cats were healthy. Based on morphology of piroplasms, the author claimed that 26% of the cats were positive for *Babesia* species and 22% for *Cytauxzoon felis*.

*Babesia canis* subspecies *presentii* was described and named from two cats in Israel; one was sick, and the other a subclinical carrier (33). The sick cat, which was coinfected with feline immunodeficiency virus and “Candidatus *Mycoplasma haemominutum*,” showed icterus and anemia. It responded rapidly to treatment with imidocarb and doxycycline and made a complete recovery.

## *Babesia* Species Reported, Based on Molecular Data

The 18S locus is a conservative marker among eukaryotes and evolves more slowly than other barcoding loci. Because of the unreliability of morphological characters for delimiting most hemoparasites, 18S is the most widely used locus to delimit hemoparasite species (34). Variation in the 18S rRNA gene has been very widely used as a taxonomic and phylogenetic tool for classifying *Babesia* species. There is, however, no universally used criterion (% sequence identity) for classifying organisms to species level based on this variation. Greay et al. (34) recently stated that the genetic distances between proposed novel apicomplexans and their closest relatives (described to date) will be greater than the genetic distances between the next two most closely related species.

The first molecular characterization of a *Babesia* species from a domestic cat was that of *B. felis* s.s., when near-full-length 18S rRNA gene sequences were entered into GenBank in 2001 (5). In this section, claims of occurrence of *B. felis* s.s. are documented and evaluated first, followed by the other *Babesia* species reported from domestic cats in alphabetical order.

### Babesia felis

The near-full-length 18S rRNA gene sequences entered into GenBank in 2001 (accession no. AF244912) are regarded as definitive for *B. felis* s.s. (5). This specimen was obtained from the isolate used for artificial infection of cats to test efficacy of various drugs in treating feline babesiosis (35, 36). Blood from three experimental cats at the Agricultural Research Council-Onderstepoort Veterinary Institute (ARC-OVI) known to harbor *B. felis* was pooled, and 4 mL injected subcutaneously into a susceptible cat. A stabilate was prepared from this cat's blood when parasitemia was approximately 12% (37); 1-mL aliquots were prepared and stored in the gas phase of a liquid nitrogen refrigerator.

One (2.9%) of 34 healthy cats in Qatar was positive for *B. felis* (99% nucleotide identity; AY452707) (38). Because there are many expatriates working in Qatar, the possibility of this cat becoming infected elsewhere cannot be ruled out.

In a study done by Salim et al. (39) to develop and optimize a loop-mediated isothermal amplification (LAMP) assay for the diagnosis of babesiosis in cats in Lahore, Pakistan, piroplasms were seen on blood smears of 45 of 100 domestic cats showing signs of anemia and lethargy. The positive samples were subjected to conventional PCR and LAMP; the LAMP assay was found to be more sensitive in detecting *Babesia* species (11/45) than the conventional PCR (5/45). The authors claimed that the piroplasms involved were *B. felis*. These finding cannot be verified, however, because close inspection of the conventional and LAMP PCR primers used in the study indicated that they could amplify any *Babesia* species Furthermore, no sequencing was done to confirm the PCR and LAMP results.

### Babesia canis

*Babesia canis* subspecies *presentii* was described from two cats in Israel (GenBank accession nos. AY272047 and AY272048) (33). The relatively short fragment (395 bp; GenBank AY15057) of the 18S rRNA gene sequenced when *B. canis* s.s. was identified in cats in Spain and Portugal (27) showed 99.5% identity when compared with the similar fragment of *B. canis presentii*. In a subsequent report from Portugal (28), specimens from 4 (1.3%) of 320 cats showed 100% identity with a *B. canis* s.s. sequence in GenBank (accession no. HQ662634.1).

### Babesia gibsoni

The only report of *B. gibsoni* in cats was from St Kitts in the Caribbean, where *B. gibsoni* was found in 5 (4%) of 119 apparently healthy cats (40). The partial 18S rRNA sequences (538 bp) found (GenBank accession no. KY073362) were identical and showed 100% identity to 37 *B. gibsoni* sequences in GenBank (e.g., accession no. JX962780).

### Babesia hongkongensis

An apparently new species, genetically and geographically distinct from other previously described *Babesia* species and tentatively named *B. hongkongensis*, was found in blood and kidney specimens of a free-ranging cat in Hong Kong (41). Its mitochondrial cytochrome b gene sequences (GenBank accession no. JQ867357) had 90.4% identity with those of *B. gibsoni* (AB499087.1). The near-full-length 18S rRNA gene sequence (1 612 bp) (GenBank accession no. JQ867356) had 96.7% nucleotide identity to various *Babesia* sequences found in feral raccoons (*Procyon lotor*) and dogs. Phylogenetic analysis of the 18S rRNA gene sequence data showed that *B. hongkongensis* falls into a distinct branch of the Babesiidae.

### Babesia lengau

*Babesia lengau* was described from cheetahs (*Acinonyx jubatus*) in South Africa (near-full-length 18S rRNA gene sequences were deposited in GenBank, accession nos. GQ411405–GQ411417) (42). Sequences for the ITS2 region were deposited in GenBank, accession numbers GQ411418 to GQ411430 (42). *Babesia lengau* was incriminated in two feline babesiosis cases in South Africa, one being the first report of cerebral babesiosis in a cat (GenBank accession nos. KC790443 and KC833036) (43).

### Babesia leo

*Babesia leo* (GenBank accession no. AF244911) was described from lions (*Panthera leo*) in Kruger National Park, South Africa (5). *Babesia leo* has been incriminated in causing disease in five cats: four in KwaZulu-Natal, South Africa and one in Maputo, Mozambique (44). Sequences from four of these cats were 100% identical to those of *B. leo*, whereas one sequence (1,520 bp) was 99% similar to *B. leo* (44).

### Babesia lohae

*Babesia lohae* (GenBank MG593272 and MG593273) was identified in a female *Ixodes holocyclus* tick recovered from a cat, as well as in an *Ixodes tasmani* collected from a brushtail possum (*Trichosurus vulpecula*) in Queensland, Australia (45). Phylogenetically, it grouped within the *Babesia* s.s. clade and with other *Babesia* species from Australian marsupials and ticks from marsupials. It is possible, therefore, that brushtail possums are a native reservoir host of *B. lohae*.

### Babesia microti

In a survey of stray cats in Milan, Italy, two (0.8%) of 260 cats tested positive for *B. microti* DNA using conventional PCR (46). Both cats were healthy. Subsequent 18S rDNA sequencing of a 261-bp fragment confirmed these two samples as *B. microti*–positive with 100% identity (over a 199 bp region) to *B. microti* previously described in *Ixodes ricinus* ticks removed from dogs in Warsaw, Poland (GenBank accession no. EU882727). In Sicily, 6 of 23 cats were PCR positive for *B. microti* (47).

One clinically affected cat in Cape Town, South Africa, was infected with both *B. felis* and *B. microti* (44). This *B. microti* (MK095343) had 100% sequence identity with *B. microti* Otsu strain (AB119446) from Japan, and 99% and 98% sequence identity with *B. microti* Gray (AY693840) and *B. microti* Munich (AB071177) strains, respectively.

In a survey in Pakistan, 21 (13.2%) of 159 cats presented at veterinary clinics were reported to be infected with *B. microti* (48). Polymerase chain reaction amplified a 238-bp amplicon specific for 18S rRNA gene of *B. microti* in which sequences were 99 to 100% identical to those deposited in GenBank (no detail given). Two partial 18S rRNA gene sequences of *B. microti* from the Pakistani cats are in GenBank (accession nos. MF401440 and MF401441). These sequences were marked as “UNVERIFIED” in GenBank (i.e., GenBank staff was unable to verify the sequences and/or annotation provided by the submitter). A BLASTn analysis showed that although MF401440 had 100% sequence identity to *B. microti* (KX758442), it had only 75% sequence coverage.

### Babesia vogeli

The first report of *B. vogeli* infection in cats was from Bangkok, Thailand, where 21 (1.4%) of 1,490 stray cats tested positive (49). Sequenced target amplicons (330 bp) from 21 samples were identical (GenBank accession no. EU697608). The authors reported that a BLASTn search indicated 98% identity with a *B. vogeli* from dogs in Brazil (GenBank accession no. not given) (50). BLASTn analysis now indicates 98.59% identity to various *B. vogeli* and *B. canis* sequences currently deposited in GenBank, but with only 86% gene coverage. Unidentified piroplasms had previously been reported from cats in Bangkok (51).

In a survey in north and central Portugal, 26 (8.1%) of 320 cats were positive for *B. vogeli*; specimens from three of these cats that were sick showed 100% identity with *B. vogeli* accession no. JX871885 in GenBank (28). In a separate study, eight cats were positive for *B. vogeli* (GenBank accession nos. AB896788–AB896795) (52). In a survey in southern Portugal, 43 (6.6%) of 649 cats were positive for *Babesia* species, possibly *B. vogeli* (53).

In Brazil, six (16%) of 37 free-ranging cats in a zoo in Sao Paolo were positive for *Babesia* (54). Their sequences (GenBank accession nos. KF970926–KF970929) (499–650 bp) showed 99% identity to *B. vogeli* (GenBank accession no. HM590440). Also in Brazil, specimens from 2 (4%) of 30 cats in Rio Grande do Sul State were positive, their sequences (GenBank accession nos. KT323932 and KT323933) (764–766 bp) showing 100% identity with *B. vogeli* (GenBank accession no. HM590440) (55).

On the island of St Kitts in the Caribbean, *B. vogeli* was found in 15 (13%) of 119 apparently healthy cats (40). All sequences were identical (GenBank accession no. KY073363) (545 bp) and showed 100% identity to 19 *B. vogeli* sequences in GenBank (e.g., accession no. HQ148664). In Qatar, one (2.9%) of 34 healthy cats was positive for *B. vogeli* (100% nucleotide identity; KT333456) (38).

### Babesia vulpes

Two clinically normal cats in Portugal that had immunosuppressive viral infections were positive for *B. vulpes* (then referred to as *Theileria annae*) (27, 56). The sequence found (AY150068) had 100% similarity with a dog isolate (AF188001). *Babesia vulpes* (referred to as *B. anna*e) was also incriminated in a clinical case in a cat in France (24). Cats were not mentioned as hosts when *T. annae* was formally renamed *B. vulpes* (57).

### *Babesia* Species Cat Western Cape

A pathogenic piroplasm from the *Babesia* s.s. clade was recently discovered in South Africa (44). Seven clinical cases were reported: six from the vicinity of Cape Town and one from Durban, KwaZulu-Natal. Because of the lack of an appropriate-type specimen, a new species was not described, and the novel organism is referred to as *Babesia* species cat Western Cape (GenBank KR611133–KR611159). All previously reported feline babesiosis cases in South Africa had been caused by *B. felis, B. lengau*, or *B. leo*.

## Discussion

In recent years, many novel *Babesia* 18S rRNA gene sequences have been deposited in the public DNA sequence databases. Apart from a limited number of 5S rRNA gene and internal transcribed spacer sequences, only 18S rRNA gene sequence data are available for the phylogenetic analysis of feline *Babesia* species of these, very few are full or near-full-length sequences; the majority are partial 18S rDNA sequence data.

In determining the phylogenetic relationships of the *Babesia* species reported from domestic cats, we used near-full-length 18S rRNA *Babesia* gene sequences available in GenBank to perform a multiple sequence alignment using ClustalX (version 1.81 for Windows) (58). None of the *B. canis, B. vogeli*, or *B. gibsoni* sequences described from cats could be included in our analysis because of the very short 18S rRNA gene fragment sizes. The alignment was truncated to the size of the smallest sequence. The Hasegawa–Kishino–Yano (HKY + G + I) substitution model (59), determined as the best-fit model using MEGA 7 (60), was used to infer a maximum likelihood phylogenetic tree. The 18S rDNA sequences of *Hepatozoon felis* (AY628681) and *C. felis* (AF399930) were included as outgroup. All positions containing gaps and missing data were eliminated. The final data set comprised a total of 1,351 positions. Evolutionary analyses were conducted in MEGA7.

**Figure 1:**
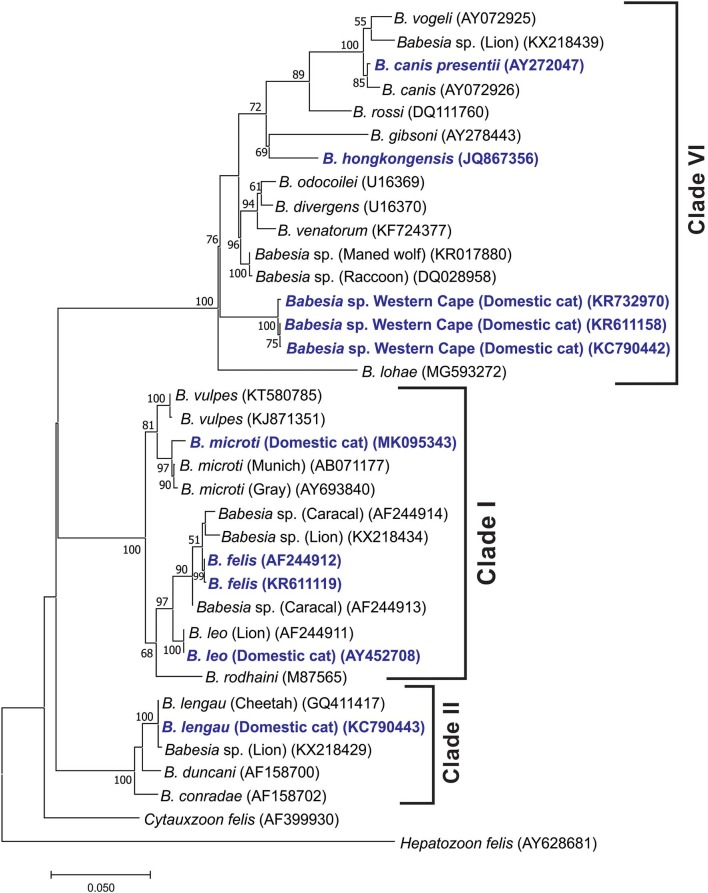
Maximum likelihood tree showing the evolutionary relationships of the published near-full-length *Babesia* 18S rDNA sequences. Babesias described from domestic cats are indicated in dark blue. Sequence accession numbers are shown in parentheses. The evolutionary history was inferred by using the maximum likelihood method based on the Hasegawa–Kishino–Yano model (HKY + G + I) substitution model (59). The tree with the highest log likelihood (−6,143.13) is shown. The percentage of trees in which the associated taxa clustered together is shown next to the branches. Initial tree(s) for the heuristic search were obtained automatically by applying Neighbor-Join and BioNJ algorithms to a matrix of pairwise distances estimated using the maximum composite likelihood approach and then selecting the topology with superior log likelihood value. A discrete gamma distribution was used to model evolutionary rate differences among sites [five categories (+G, parameter = 0.4871)]. The rate variation model allowed for some sites to be evolutionarily invariable [(+I), 55.35% sites]. The tree is drawn to scale, with branch lengths measured in the number of substitutions per site. All positions containing gaps and missing data were eliminated. There were a total of 1,351 positions in the final data set. Evolutionary analyses were conducted in MEGA7 (60).

The *Babesia* species from cats fell into three distinct clades (Figure 1), in concordance with Schnittger et al. (61). Clade I also includes rodent-infecting *B. microti* and *Babesia rodhaini* and feline-infecting *B. leo* and *B. felis*. Clade II also includes *Babesia duncani* isolated from humans, canine *Babesia conradae*, and *B. lengau* described from cheetahs in South Africa. The recently described pathogenic *Babesia* species cat Western Cape (44) fell into clade VI (*Babesia* s.s.). This clade also includes dog-infecting *B. gibsoni, B. canis* s.s., *Babesia rossi*, and *B. vogeli*, the human-infecting *Babesia venatorum*, as well as species infecting ungulates (such as *B. divergens* and *Babesia odocoilei*), and *Babesia* species infecting other carnivores such as bears, cougars, and raccoons, as well as field rodents.

Our analysis placed *B. hongkongensis* in clade VI [also referred to as the “carnivore/rodent clade” by Schnittger et al. (61)], confirming the findings of Wong et al. (41). The feline genotype of *B. canis* described as a new subspecies *B. canis presentii* (33), grouped with *B. canis* within the *Babesia* s.s. clade. *Babesia lohae* grouped within the *Babesia* s.s. clade as described by Greay et al. (45). *Babesia vulpes* grouped within the *B. microti* group.

**Figure 2:**
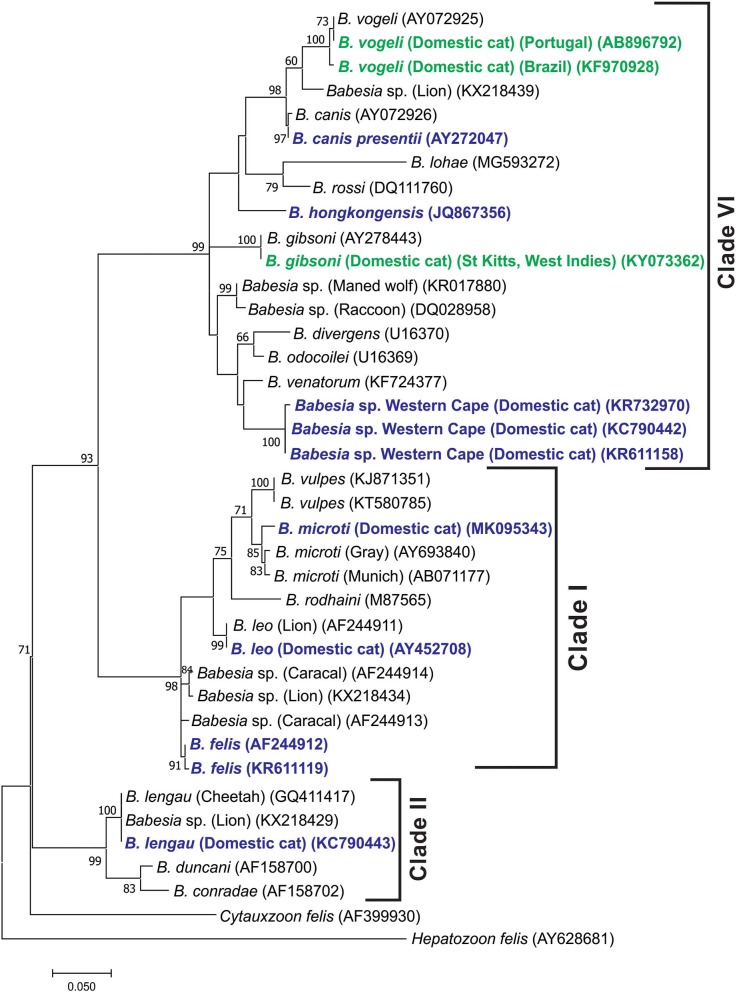
Molecular phylogenetic analysis showing relationships between representative *B. vogeli* and *B. gibsoni* partial 18S RNA gene sequences described from cats with the published *Babesia* 18S rDNA sequences. Babesias described from domestic cats are indicated in dark blue. The *B. vogeli* and *B. gibsoni* sequences described from domestic cats are indicated in dark green. Sequence accession numbers are shown in parentheses. The evolutionary history was inferred by using the maximum likelihood method based on the Tamura 3-parameter model (62). The tree with the highest log likelihood (−1,898.16) is shown. The percentage of trees in which the associated taxa clustered together is shown next to the branches. A discrete gamma distribution was used to model evolutionary rate differences among sites [five categories (+G, parameter = 0.3970)]. The rate variation model allowed for some sites to be evolutionarily invariable [(+I), 38.96% sites]. The tree is drawn to scale, with branch lengths measured in the number of substitutions per site. All positions containing gaps and missing data were eliminated. There were a total of 312 positions in the final data set. Evolutionary analyses were conducted in MEGA7 (60).

When a phylogenetic tree was generated from partial 18S rDNA sequence data (312 bp) to include the *B. canis* s.s., *B. vogeli*, and *B. gibsoni* sequences described from cats, similar groupings were found, although the branching of the clades was slightly different (Figure 2). Note that the *B. canis* sequences identified in cats in Spain and Portugal (27) could not be found in GenBank under the accession no. AY15057 and were therefore not included in the analysis.

The four *Babesia* species known to cause disease in cats in South Africa fall in three distinct clades (61). The closely related *B. felis* s.s. and *B. leo* fall in clade I, *B. lengau* in clade II, and *Babesia* species cat Western Cape in clade VI (*Babesia* s.s.). In retrospect, differences in clinical manifestation, for example, presence or absence of pyrexia and response to specific treatment, reported in South African literature (1937 to 1980) suggest that more than one *Babesia* species was involved.

The dog-associated *B. vogeli* appears to be quite widespread in domestic cats, with reports from Brazil (54, 55) and St Kitts (40) in the Americas, Portugal (28, 52) in Europe, Qatar in the Middle East (38), and Thailand (49) in Asia. This is not surprising because its vector, *Rhipicephalus sanguineus* s.l., has a cosmopolitan distribution. Its relatively high prevalence in clinically healthy cats, for example, 16% in Brazil (54), 13% on St Kitts (40), and 8.1% in Portugal (28), suggests that immunocompetent cats can harbor the infection with no discernible untoward effects. None of 27 cats tested in South Africa was positive for *B. vogeli* (63). The European *Dermacentor reticulatus*–transmitted *B. canis* s.s., on the other hand, was found in only 4 (1.3%) of 320 cats in north and central Portugal (28).

*Babesia rossi*, which causes severe babesiosis in dogs in sub-Saharan Africa, has not been reported from cats. Specimens from 27 cats tested specifically for *B. rossi* were negative (63). Since *Haemaphysalis elliptica*, the only known vector of *B. rossi*, is the most prevalent and abundant tick on cats in the Western Cape Province of South Africa (64), where canine babesiosis is rife, it is not unlikely that *B. rossi* may be recorded in local cats. Three separate attempts at artificial transmission of *B. rossi* from dogs to cats were unsuccessful, however (65–67).

## Conclusion

In the absence of easily discernible morphological differences, molecular characterization is indispensable in distinguishing between *Babesia* species and in identifying and describing new taxa. Molecular characterization has made it abundantly clear that referring to all piroplasms encountered in cats merely as *B. felis* is no longer acceptable. In view of the body of literature on the topic published since 2001, persisting in doing so is scientifically irresponsible. Reports of occurrence of *B. felis* s.s. and other *Babesia* species in domestic cats should only be accepted if they are supported by credible molecular provenance.

## Author Contributions

BP performed the literature searches and drafted the basic manuscript. MO performed the phylogenetic analyses and wrote the relevant sections.

### Conflict of Interest

The authors declare that the research was conducted in the absence of any commercial or financial relationships that could be construed as a potential conflict of interest.
